# When Should an External Urethrotomy Be Done

**Published:** 1910-11

**Authors:** T. M. McIntosh

**Affiliations:** Thomasville, Ga.


### Journal-Record of Medicine

Successor to Atlanta Medical and Surgical Journal, Established 1855, and Southern Medical Record, Established 1870.

OWNED BY THE ATLANTA MEDICAL JOURNAL CO.

Published Monthly

*Official Organ Fulton County Medical Society, State Examining Board, Presbyterian Hospital, Atlanta, Birmingham and Atlantic Railroad Surgeons' Association, Chattahoochee Valley Medical and Surgical Association, Etc.*

EDGAR G. BALLENGER., M. D., Editor.
BERNARD WOLFF, M. D., Supervising Editor.
A. W. STIRLING, M. D., C. M., D. P. H., J. S. HURT, B. Ph., M. D.
GEO. M. NILES, M. D., W. J. LOVE, M. D., (Ala.); Associate Editors.
E. W. ALLEN, Business Manager.

COLLABORATORS

Dr. W. F. WESTMORLAND, General Surgery.
F. W. McRAE, M. D., Abdominal Surgery.
H. F. HARRIS, M. D., Pathology and Bacteriology.
E. B. BLOCK, M. D., Diseases of the Nervous System.
MICHAEL HOKE, M. D., Orthopedic Surgery.
CYRUS W. STRICKLER, M. D., Legal Medicine and Medical Legislation.
E. C. DAVIS, A. B., M. D., Obstetrics.
E. G. JONES, A. B., M. D., Gynecology.
R. T. DORSEY, Jr., B. S. M. D., Medicine.
L. M. GAINES, A. B., M. D., Internal Medicine.
J. N. LeCONTE, M. D., Disease of the Stomach and Intestines.
L. B. CLARKE, M. D., Pediatrics.
EDGAR PAULIN, M. D., Opsonic Medicine.
THEODORE TOEPEL, M. D., Mechano Therapy.
R. R. DALY, M. D., Medical Society.
A. W. STIRLING, M. D., etc., Diseases of the Eye, Ear, Nose and Throat.
BERNARD WOLFF, M. D., Diseases of the Skin.
E. G. BALLENGER, M. D., Diseases of the Genito-Urinary Organs.

VOL. LVI. NOVEMBER, 1910 NO. 8.

### WHEN SHOULD AN EXTERNAL URETHROTOMY BE DONE.

T. M. McINTOSH, THOMASVILLE, GA.

The most frequent conditions requiring an External Urethrotomy is the result of neglected strictures of the urethra, following specific Urethritis, and the complications resulting therefrom, as impermeable stricture, perineal abscess, old and numerous urinary fistula, acute retention of urine, and when false passages and pockets have been made in the urethra, in ineffectual attempts to enter the bladder, by the use of improper and too rigid instruments. Also to procure drainage of the bladder, and immediate rest to that organ, in acute, and obstinate chronic cystitis, accompanied with persistent, painful, and exhausting vesical tenesmus, and calculi lodged in any part of the urethra.

To illustrate clearly the different conditions which call for an external urethrotomy, it will suffice to briefly outline, in a general way, cases in which it has been necessary to resort to this operation. In concluding remarks it will not be out of place, though not strictly in harmony with the text of this article, to make a few general remarks as to how, in the treatment of the advanced conditions of disease which may demand External Urethrotomy, one should best proceed in order to avoid this operation, for certainly a resort to an external urethrotomy is necessary, in most instances, only because of delayed and improper treatment of the conditions which require this operation. Usually the initial symptoms are disregarded by the patient, either from negligence, ignorance or indifference, for they consult the physician only when the symptoms are so painful and urgent as to compel a resort to surgical treatment; or sometimes, beginning treatment sufficiently early, it is discontinued as soon as the symptoms cease to be annoying or inconvenient.

Case 1.—Mr. H., Decatur Co., farmer, married. Aged about 25 years, large, well-muscled man, of good family and personal history. Never had any venereal disease. Had suffered for a year when he came under my care, with frequent, painful urination, great vesical tenesmus, and much mucus and some blood at times in urine.

There was no stricture, no stone in bladder, no disease of the kidney. The interior of the bladder could not be visually examined, as at that time instruments for such examinations were not in use.

The usual treatment of saline and vegetable diuretics, with washing of the bladder, was given for six months, with no permanent relief or abatement of symptoms. An external (perineal) urethrotomy was done, to procure vesical rest by continuous drainage. The bladder was explored with the finger, and no discoverable lesion or growth found to account for the cytitis· A soft rubber drain was put into the bladder and retained for one week, after which time the wound was kept open for the daily passage of a soft rubber Bongie. The relief was prompt and complete, as long as the wound was kept open. When it closed there was a partial recurrence of the symptoms, but greatly modified; but he grew gradually better, and in six months he was nearly well. The cause could not be definitely found.

Case 2.—Was asked by one of my colleagues to go in the country, to see and operate on James C., a negro man, about 30 years old. A history of previous Gonorrhea, and difficulty of urination, for some time past. There was fever. The entire perineum was swollen, and fluctuation by palpitation was marked. The swelling extended up into the groin, into the lower abdominal walls on either side, and in the right Inguimal region was a spontaneous opening, discharging pus. He was passing urine with difficulty through urethra. The perineum was incised from the scrotum to the anus, giving exit to a large amount of pus and putrid urine. The left side of the abdominal wall was opened, giving exit to pus. The abdominal wall, and perineum openings were freely syringed with warm, carbolized water. Later the stricture of urethra which caused the abscess was treated. The pus in this case had not ulcerated through the superficial perineal fascia and discharged there, but had followed the reflection of that fascia to the groin and into the abdominal fascia, the fascia of those regions being continuous.

Case 3.—John B., colored, Brooks Co., aged 30. History of Syphilis and Gonorrhea. Had multiple urinary fistulae. The posterior fistula being near the anus, the anterior one being anterior to the scrotum, and between these, numerous other fistulas. Through them all, urine dripped at each urination. It was not possible to pass a filiform through this long stretch of diseased urethra into the bladder. An incision in the median line was made between the anterior and posterior fistula, opening the urethra the entire distance, and cutting through a mass of indurated tissue throughout the incision. A soft rubber catheter was passed from the meatus to the bladder, tied, and retained in sitee for two weeks, It was then withdrawn and passed daily for two more weeks. The patient was instructed now to use the bongie, and sent home. While the catheter was in the bladder, the outer end was stoppered, and when patient wished to urinate, the stopper was withdrawn for the urine outflow.

Case 4.—Mr. F. F., Gadsen Co., Fla., age 40. Had been treated by gradual dilatation of an old stricture. Ceased treatment, and urethra closed. Came to me suffering with violent efforts to urinate, the urine coming drop by drop. High fever, great pain. After an hour's fruitless effort to pass a filiform into the bladder, the urethra was opened by external incision. No catheter for drainage was used; but after the fever subsided daily introductions of bongies were made.

Case 5.—Mr. J. G., Madison, Fla. Was called by telegram to relieve a case of "retention from stricture." Arrived at night, found a young man, with a previous history of Gonorrhea, with some difficulty of urination for some weeks past. There was high fever, great vesical pain, and intense effort to urinate. Persistent and ineffectual attempts had been made to catheterize the bladder, and this had produced considerable injury to and bleeding from the urethra. I made no attempt to pass an instrument into the bladder, but proceeded at once to make an external urethrotomy by lamp light and without a guide.

Case 6.—Thos. B., col., aged 65, Early Co. Gave a history of frequent and painful urination for a year or so. At times the urine came free, and then would suddenly stop, with great vesical tenesmus. The urine at the time he came to me was voided with much effort, and the bladder never completely emptied, as evidenced by palpitating over the abdomen, outlining the distended bladder. There was a firm swelling in the perineum. A number six soft rubber catheter was passed easily to the point of swelling, and obstructed. A small metal bongie was then passed and a stone detected lodged in the membranous portion of the urethra. This was removed by external incision. The stone was half an inch long, the size of a lead pencil and symmetrically oblong.

In a three year old child, with painful, frequent and obstructed urination, a small metal bongie was passed into the urethra to discover the cause of obstruction, and a stone was detected just anterior to the bulb. Under anaesthesia, the largest metal bongie that the urethra could possibly admit, was passed down to the stone. A finger was pressed against the urethra behind the stone, thus fixing the stone between the bongie and the finger. The bongie was slowly withdrawn, at the same time pressing the stone with the finger, behind the receding bongie, the stone was gradually forced to follow the bongie, and thus delivered.

If a stricture is of such small caliber, that a number ten French scale, bongie, cannot readily pass into the bladder, any metal, or gum instrument, made rigid by a stylet, should never be used, because of the almost certainty of making a pocket or a false passage in the urethra. In such a narrowed urethra, one should use progressively smaller numbers of soft cone-pointed silk or gum instruments,—never a rigid bongie and never use any force. If none of these bongies can pass the stricture, then the filiform must be used, beginning with the smallest and finest of these. If any filiform catches in a pocket or fold of tissue, insert another along side this, and then another and another, till you have five or six in the urethra at the same time. Then withdraw slightly one of the filiform and then push it gently deeper. If it does not enter the narrowed passage and go into the bladder, leave that bongie alone for the time, and go through with the same proceedings again and again with all the other bongies then in the urethra, continuing and repeating this procedure, gently, never using force, for an hour or more, and in the great majority of cases, you will enter the bladder with one of the filiforms. The bladder entered by one filiform, remove them all, but the one in the bladder. By filling the urethra in this manner, full of filiform bongies, you distend the urethra above the stricture, and form a protecting wall of whale-bone bongies, which prevents making a false passage or a pocket in the urethral wall.

The bladder once entered, you are master of the situation, and can take your time as to the insertion of a large instrument. If it be a case of acute retention, it will not be necessary to pass into the bladder a tunnelled catheter, with the filiform as a guide, for a tied and left-in filiform will so open the urethral canal at the point of stricture, that urine will pass around the filiform; and if it be a close, hard stricture, it will so soften the tissue, within twelve to twenty-four hours, that you can pass a slighlty larger cone-pointed gum or silk catheter, and these in turn can be tied and left in the urethra, which again will have a softening and dilating effect on the toughest submucus stricture tissue. Of course the patient this while is in bed.

By patient, persisting, and deliberate efforts to enter the bladder through a close stricture, after the manner above outlined, one will be able to succeed without injury to the urethra, and without causing such general disturbances as urethra chill, and resulting high fever, and thus avoid an external urethrotomy, and while this operation is not a major one, yet the after treatment is more unpleasant and prolonged, and your patient will be much better pleased if a cure is made without a cutting operation, and with a shorter time in bed.

Two years ago, a prominent N. Y. surgeon, an author, attempted to pass a filliform bongie into the bladder, through a close stricture. The patient was passing urine and had no fever. Not succeeding as readily as he anticipated, he became a little nervous, and with a flushed face, made an extensive external urethrotomy. A little more time and a little more patience would have enabled him to accomplish what he had planned to do, and his patient would have been cured in a shorter time of his stricture.

It will be observed that in those cases when there was acute retention of urine, great vesical pain and with high fever, indicating grave constitutional disturbance, that an external urethrotomy was made, with but little effort to pass an instrument of any kind into the bladder, for under such conditions, efforts to pass instruments will cause more or less additional irritation to the urethra, and thus intensify the febrile disturbance, and add to probabilities of urethral chill, which is a very grave complication, and may lead to serious results.

An external urethrotomy relieves at once the bladder, and afterward the urine passing freely through the incision, further instrumentation is not immediately necessary, and can be deferred until the febrile symptoms have gone, when treatment for cure of the stricture can be done, usually best by gradual dilatation and with conical pointed soft bongies, up to a number ten French, after which metal bongies may be used and carried up to as high a number as each case may demand. Rigid instruments of small size are not proper to use in any close stricture of the urethra, as you are liable to do harm with them. In any case of stricture, when urine is passing, if there be fever, or the patient is in low condition, any urethral instrumentation is attended with risk, and as little should be done as possible, and only for the immediate comfort of the patient. The year after my graduation, a young professor of surgery brought a negro man into the amphitheater to operate for urethral stricture. A few days before, an acute retention had been relieved, and he was passing his urine. There was high fever, following the chill resulting from the catherization that relieved the retention. An internal urethrotomy was made before the class, and the usual after introduction of a good-sized metal bongie.

The same day there was another urethral chill, and the patient was lost.

An external urethrotomy when demanded, gives pleasing results. Too much urethral instrumentation on patients with febrile disturbance and in enfeebled general condition, had best be avoided as much as possible.

